# Changes in the Microbiome in Yak Mastitis: Insights Based on Full-Length 16S rRNA Sequencing

**DOI:** 10.3390/vetsci11080335

**Published:** 2024-07-25

**Authors:** Lihong Zhang, Hongcai Ma, Wenqiang Tang, Jiangyong Zeng, Md. F. Kulyar, Junjie Hu

**Affiliations:** 1 College of Veterinary Medicine, Gansu Agricultural University, Lanzhou 730070, China; hujj@gsau.edu.cn; 2 Institute of Animal Husbandry and Veterinary Medicine, Xizang Academy of Agriculture and Animal Husbandry Sciences, Lhasa 850009, China; xzmahongcai@126.com (H.M.); tang.iwate@gmail.com (W.T.); zengjiangyong@126.com (J.Z.); 3 College of Veterinary Medicine, Huazhong Agricultural University, Wuhan 430070, China; fakharealam786@hotmail.com

**Keywords:** mastitis, mammary gland, probiotics, 16S rRNA sequencing, yak

## Abstract

**Simple Summary:**

Mastitis is an infection of the mammary glands that affects yaks‘ health and milk quality. The various bacteria found in mastitis-affected yaks were examined using a technique for reading particular genetic information. We discovered that contaminated milk had higher concentrations of bacteria than healthy milk. Our findings highlight the complexity and irregularity in the milk microbiome of yaks. Understanding these bacterial communities may aid in developing more effective mastitis control measures and enhance the health and milk quality of yaks, thereby benefiting dairy producers and yak farmers.

**Abstract:**

Mastitis is an inflammation of the mammary gland that can be caused by various factors, including biological, chemical, mechanical, or physical. Microbiological culture, DNA techniques, and high-throughput next-generation sequencing have been used to identify mastitis-causing pathogens in various animal species. However, little is known about microbiota and microbiome changes linked to yak milk mastitis. This study aimed to characterize the milk microbiota of healthy and mastitis-infected yaks using full-length 16S rRNA sequencing. The results showed that the bacterial microbiota comprises 7 phyla, 9 classes, 20 orders, 39 families, 59 genera, and 72 species. *Proteobacteria* and *Firmicutes* were the predominant microbial communities, with lower abundances of *Bacteroidota*, *Actinobacteriota*, *Acidobacteriota*, and other minor groupings also observed. *Proteobacteria* dominated the clinical and subclinical mastitis groups (95.36% and 89.32%, respectively), in contrast to the healthy group (60.17%). Conversely, *Firmicutes* were more common in the healthy group (39.7%) than in the subclinical and clinical mastitis groups (10.49% and 2.92%, respectively). The predominant organisms found in the healthy group were *Leuconostoc mesenteroides*, *Lactococcus piscium*, *Carnobacterium maltaromaticum*, and *Lactococcus raffinolactis*. Low abundances of *Staphylococcus aureus* species were found in both subclinical and clinical mastitis groups, with *Moraxella osloensis* and *Psychrobacter cibarius* dominating the subclinical mastitis group and *Pseudomonas fluorescens* dominating the clinical mastitis group. An alpha diversity study revealed that the healthy group had a higher microbial diversity than the clinical and subclinical mastitis groups. According to beta-diversity analysis, the principal coordinate analysis identified that mastitis-infected samples significantly differed from healthy ones. The milk microbiota of healthy yaks is more varied, and specific prominent taxa within various groups can act as marker microorganisms for mastitis risk. The genera *Leuconostoc* and *Lactococcus* are promising candidates for creating probiotics.

## 1. Introduction

Mastitis is a well-known inflammatory condition affecting dairy cattle udders and significantly affecting the quantity and quality of milk dairy herds produce and their general well-being [1]. Numerous bacteria that enter the teat canal and cause inflammation and harm to the mammary gland tissue are the primary triggers of this disorder [2]. Mastitis typically induces pain and discomfort in affected cows, which reduces their feed intake and milk output [3,4]. As a result, it is critical to understand the substantial impact of this condition on milk production. Additionally, changes in the somatic cell counts (SCC) and disruptions in the homeostasis of the bovine milk microbial community may indicate infection in the udders, resulting in modifications to the physical and chemical properties of the milk [5,6]. It becomes evident that mastitis poses significant challenges for dairy farmers worldwide due to its adverse effects on milk production levels and the long-term viability of their businesses.

Molecular methods have emerged as state-of-the-art technology for disease diagnosis, exceeding conventional methods due to the rapid advancement of high-throughput sequencing techniques in recent years. These techniques have demonstrated outstanding efficiency and dependability while offering a comprehensive knowledge of various microbes in complex ecosystems, including the udder microbiome [7,8]. Several microbiological techniques have identified specific bacterial taxa linked to healthy, subclinical, and clinical mastitis. Without the requirement for isolation or cultivation, these investigations have made it possible to identify every bacterium found in a sample fully and have contributed to understanding microbial communities’ functional profiles [9,10]. A comprehensive review of research conducted on milk microflora shows that typical taxa with various geographical origins are reliably identified in cows’ milk. In studies on the microbiota of bovine dairy products, *Streptococcus*, *Pseudomonas*, *Bifidobacterium*, *Propionibacterium*, *Bacteroides*, *Corynebacterium*, and *Enterococcus* are the leading taxa that are most frequently reported [11]. Furthermore, it has become easier to monitor changes in the populations of pathogenic bacteria that cause mastitis infections in cattle by applying metataxonomic approaches [12,13]. These findings contribute to understanding the interactions between bacteria in this environment and their impact on mammary gland health. Therefore, an in-depth understanding of the microbial composition of the udder microbiome is essential in preventing or curing this illness.

Yaks are an indigenous breed with properties enabling them to thrive in the harshest environments [14]. Yaks are significant to the local economy and culture due to their milk, meat, fiber (yak wool), and mobility [15]. The nutrient-rich milk of yaks is a vital nutritional resource for local populations who rely on this versatile animal for dairy products such as butter, cheese, yogurt, and traditional beverages [16]. However, little is known about the microbiota of its milk or udder. In this work, we comprehensively investigated bacterial microbiota based on full-length 16S rRNA sequencing to investigate yaks’ milk microbiota. By identifying different taxa of bacteria associated with healthy and mastitis-affected yaks, we intend to develop specialized probiotics that can effectively prevent or treat yak mastitis.

## 2. Materials and Methods

### 2.1. Sample Collection and Assessing Clinical Health Status

A total of 235 yak milk samples were collected in the Naqu district of Xizang. Based on comparable husbandry and management techniques, four homes were carefully chosen to guarantee the study’s validity and dependability. To ensure consistency within our sample population, we included only mature yaks between the ages of three and six that had undergone at least one lactation phase and had not received antibiotic treatment in the previous two months. The animals were separated into healthy and mastitis groups based on physical examination, assessment of udder condition, Lanzhou mastitis test (LMT), and somatic cell counts in milk samples. Out of all the samples collected, fifteen were chosen at random and taken for analysis. The samples included five from animals in good health with no signs of abnormal udder health, five from those with clinical mastitis and apparent abnormalities or inflammation, and five from those with subclinical mastitis showing no symptoms but still having a bacterial infection detectable through laboratory testing. Following the laboratory and field handbook instructions on bovine mastitis, milk samples were collected from each of the chosen animals as part of the mastitis detection screening procedure. The original milk was disposed of, and the teat-ends were carefully cleansed and properly sanitized with a 70% ethanol solution before being collected. The milk samples were transported to the laboratory and kept at −80 °C until further processing. 

### 2.2. DNA Extraction

The DNA was extracted from yak milk samples using the TGuide S96 Magnetic Soil/Stool DNA Kit (Tiangen Biotech Co., Ltd., Beijing, China) following the manufacturer’s instructions. The DNA content of the samples was determined using the Qubit dsDNA HS Assay Kit and Qubit 4.0 Fluorometer (Invitrogen, Thermo Fisher Scientific, Hillsboro, OR, USA).

### 2.3. Amplicon Sequencing 

The full-length 16S rRNA gene was amplified from the isolated genomic DNA of each sample using the universal primer sets 27F (AGRGTTTGATYNTGGCTCAG) and 1492R (TASGGHTACCTTGTTASGACTT). Sample-specific PacBio barcode sequences were incorporated into the forward and reverse primers to facilitate multiplexed sequencing. Barcoded primers were preferred over an alternative method involving a second PCR reaction to lessen the production of chimeras. The amplification procedure included 25 cycles of PCR using KOD One PCR Master Mix (TOYOBOLife Science, Tokyo, Japan), with the initial denaturation occurring at 95 °C for two minutes. Following this, denaturation occurred for 10 s at 98 °C, 30 s of annealing at 55 °C, 1 min and 30 s of extension at 72 °C per cycle, and an additional 2 min of annealing at 72 °C. After the PCR, amplicons were purified using Agencourt AMPure XP Beads (Beckman Coulter, Indianapolis, Indiana, USA), the findings were quantified using the Qubit 4.0 Fluorometer and dsDNA HS Assay Kit (Invitrogen, Thermo Fisher Scientific, Hillsboro, OR, USA). The exact number of amplicons was merged after each individual was quantified. The amplified DNA was used to build SMRTbell libraries using the SMRTbell Express Template Prep Kit 2.0 and the manufacturer’s instructions (Pacific Biosciences, Menlo Park, CA, USA). The purified SMRTbell libraries from the pooled and barcoded samples were sequenced on a single PacBio Sequel II 8M cell using the Sequel II Sequencing Kit 2.0.

### 2.4. Bioinformatic Analysis

The bioinformatics analysis of this work was performed using the BMK Cloud (Biomarker Technologies Co., Ltd., Beijing, China) to enhance its computing capabilities. The raw sequencing reads were filtered and demultiplexed using SMRT Link software (version 8.0; https://www.pacb.com/smrt-link/; accessed on 18 January 2024), with specific requirements minPasses ≥ 5 and minPredictedAccuracy ≥ 0.9, to obtain high-quality circular consensus sequencing (CCS) reads. Next, based on barcodes, the CCS reads were assigned to the proper samples using Lima software (version 1.7.0). To perform quality filtering and identify forward and reverse primers, CCS values that were either primer-deficient or fell outside the 1200–1650 bp length range were removed using the Cutadapt (version 3.4) quality control process [17]. Using the UCHIME method (version 8.1; https://drive5.com/usearch/manual/uchime_algo.html; accessed on 1 February 2024) to identify and eliminate chimeric reads produced high-quality readings. Reads showing ≥97% similarity were classified into the same operational taxonomic unit (OTU) using USEARCH ￼ (version 10.0), and OTUs with an abundance of <0.005% were filtered out. The taxonomy annotation of the OTUs was performed with a 70% confidence level based on the naive Bayes classifier in QIIME2 [18] using the SILVA database [19]. The programs used to calculate and show the alpha diversity were R and QIIME2, respectively. Using QIIME, beta diversity was assessed to gauge how similar the microbial communities were across different samples. Principal coordinate analysis (PCoA), heat maps, unweighted partial graph modeling (UPGMA), and nonmetric multidimensional scaling (NMDS) were employed in the beta diversity analysis. Furthermore, we used linear discriminant analysis (LDA) effect size (LEfSe) to look for significant taxonomic differences across groups. A logarithmic LDA score of 4.0 was used as a threshold for discriminative features. Redundancy analysis (RDA) was used to examine the variations in microbiome composition associated with different parameters. The package vegan in R (version 3.1; https://cran.r-project.org/web/packages/vegan/index.html; accessed on 18 February 2024) was utilized for this purpose.

### 2.5. Statistical Analysis

The data were analyzed using a one-way analysis of variance (ANOVA) and the least significant difference (LSD) test in SPSS 22.0. The information was provided as the standard error (SEM) ± mean. The statistical significance levels were set at *p* < 0.05 or *p* < 0.01 to show significant or extremely significant differences between the groups. Utilizing the resources available on the Biomarker Technologies Co., Ltd. (Beijing, China) BMKCloud platform (www.biocloud.net; accessed on 3 March 2024), we investigated alpha/beta diversity indices, Venn diagrams, and classes of bacteria.

## 3. Results

### 3.1. Somatic Cell Counts

Our results show that 86 (36.6%) of the 235 yaks whose udder health was assessed had subclinical mastitis but they showed no abnormalities or inflammatory signs. Still, these yaks exhibited SCC > 200,000 cells/mL milk and tested positive for LMT. Following the National Mastitis Council requirements, only 10 (4.25%) instances had mammary gland irritation with obvious inflammatory signs in the udder or anomalies in the milk.

### 3.2. Taxonomic Identification of Milk Bacterial Community in Yak 

**Figure 1:**
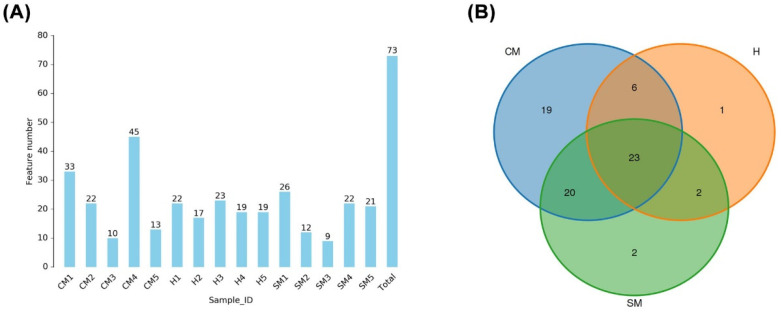
Operational taxonomic units (OTUs) of different samples. (**A**) Distribution of OTU numbers within each group and sample. (**B**) OTU Venn diagrams for the three groupings.

We used 16S rRNA sequencing to evaluate the bacterial community’s diversity within each sample and group. All of the collected yak milk samples had 193,645 CCS reads that were obtained using the PacBio platform. The 193,191 high-quality, effective CCS reads were obtained after read-quality filtering; the total number of reads for the clinical mastitis, subclinical mastitis, and healthy groups were, respectively, 12,999 ± 243, 12,671 ± 471, and 12,967 ± 267 (Table S1). Based on a 97% sequence similarity, the average number of OTUs in all samples was 21 (range 9 to 45, SEM = 2). The rarefaction curves on the number of OTUs show that the sequencing depth utilized in this study sufficiently described the bacterial microbiota of the milk samples (Figure S1). The Venn diagram (Figure 1) shows that all three groups shared 23 OTUs; the groups with clinical mastitis, subclinical mastitis, and healthy skin had 1, 2, and 19 distinct OTUs, respectively. The examination of the milk microbiome profile revealed a variable distribution of 7 phyla, 9 classes, 20 orders, 39 families, 59 genera, and 72 species among numerous groups.

### 3.3. Characterization of the Bacterial Diversity in Yak Milk Samples

**Figure 2:**
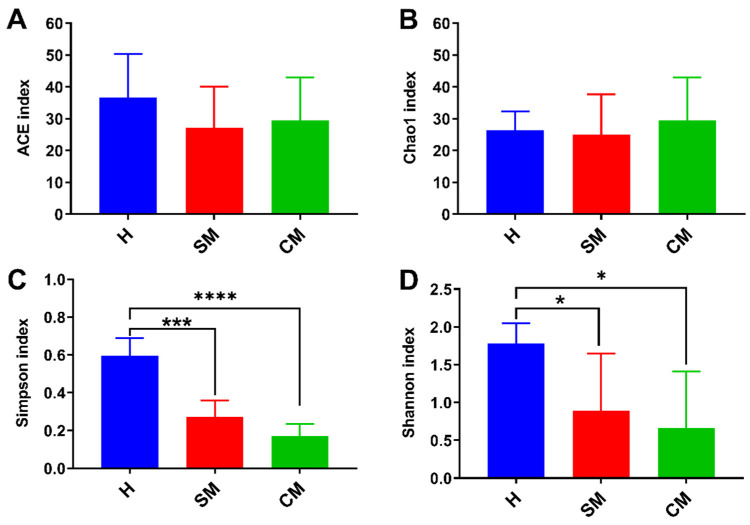
The microbiota alpha diversity was measured using the ACE (**A**), Chao1 (**B**), Simpson (**C**), and Shannon (**D**) Indices. Significant differences with *p* < 0.05 are indicated by an asterisk (*); highly significant differences with *p* < 0.001 are marked by three asterisks (***) and p < 0.00001 are marked as (****). H: healthy group, SM: subclinical mastitis group, CM: clinical mastitis group.

**Figure 3:**
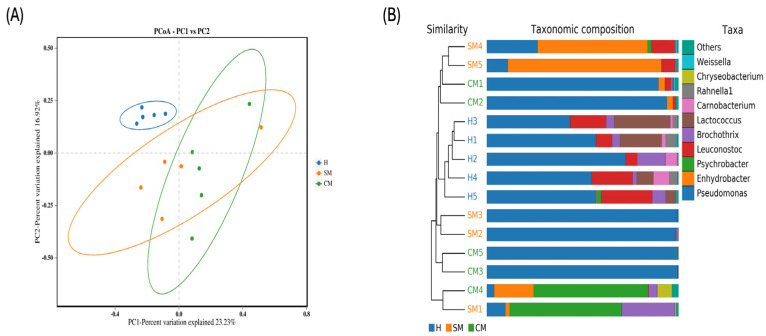
Microbiota beta diversity in the yak milk of the healthy (H), subclinical mastitis (SM), and clinical mastitis (CM) groups was assessed using. (**A**) PCoA score plot. (**B**) Binary-Jaccard method with arithmetic mean (UPGMA) analysis.

The α and β-diversity indices (ACE, Chao1, Shannon, and Simpson) and binary Jaccard, Bray–Curtis, unweighted, and weighted Unifrac metrics were used to compute the microbiota diversity values for the three groups. Alpha diversity is indicative of the richness and diversity of the microbiota. Comparisons between the Shannon and Simpson index results of the healthy group (H) and clinical mastitis (CM) group and between the healthy group and subclinical mastitis (SM) group both showed a significant difference (*p* < 0.001 and *p* < 0.05, respectively). The ACE and Chao1 indices did not significantly differ from one another in any of the comparisons (Figure 2). In addition; the β-diversity was measured using binary Jaccard PCoA to evaluate the similarities between microbial communities. All milk samples were found to be grouped into three clusters (Figure 3), with principal components PC1, PC2, and PC3 accounting for 23.23%, 16.92%, and 14.25% of the observed variance, respectively. These findings were based on Adonis [20], *p* = 0.001, R^2^ = 0.486. UPGMA analysis supported the findings, demonstrating that the clinical mastitis group matched the subclinical mastitis group more closely than the healthy group.

### 3.4. Taxonomic Bacterial Profiling of Milk Samples from Yak

**Figure 4:**
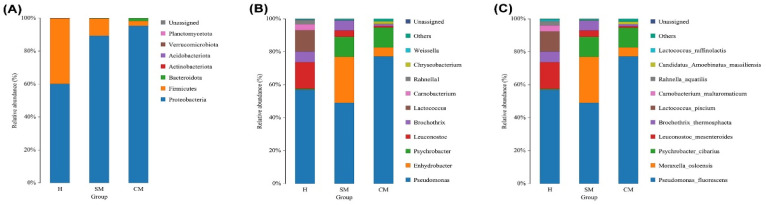
The relative abundance of the milk bacterium community for healthy (H), subclinical (SM), and clinical mastitis (CM) groups. (**A**) Phylum; (**B**) genus; (**C**) species.

*Proteobacteria* accounted for 81.61% of the most predominant phylum, followed by *Firmicutes* (17.71%). In lesser abundances, *Bacteriodota*, *Actinobacteriota*, *Acidobacteriota*, and more small groups were found (Figure 4A). *Predominant* was most prevalent in the group with clinical mastitis (95.36%), followed by the subclinical mastitis group (89.32%), and the healthy group (60.17%). Most *firmicutes* were observed in the subclinical mastitis group (10.49%) and the healthy group (39.7%). It was identified in lower abundance (2.92%) in the group with clinical mastitis. The clinical mastitis group had the highest abundance of *Bacteroidota* (1.69%), followed by the subclinical mastitis group (0.14%), whereas the healthy group had the lowest (0.005%). *Actinobacteriota* was more abundant in the healthy group (0.057%) than in the clinical mastitis group or subclinical mastitis groups. *Acidobacteriota* was only discovered in the group with clinical mastitis; it was not present in the other groups (Table S2).

The 59 taxa were identified overall by taxonomic classification of the yak milk microbiome, with varying abundance levels in each of the three groups. The *Pseudomonas*, *Leuconostoc*, and *Lactococcus* genera were found to be highly abundant in the healthy group (Figure 4B). There was an indication of *Pseudomonas* in all three groups. However, the clinical mastitis group had the highest percentage abundance (77.18%), followed by the subclinical mastitis group (49.03%), and the healthy group (56.93%). The subclinical mastitis group had the significantly highest abundance of the *Enhydrobacter* genus (27.94%) compared to the other groups. In terms of percentage abundance, the subclinical mastitis group had the highest percentage abundance of the *Psychrobacter* genus (12.15%), followed by the clinical mastitis group (11.95%). In comparison, the healthy group (0.52%) had the lowest. The *Leuconostoc* genus was discovered to be the most common (16.14%) in the healthy group, with a considerable abundance. This was followed by the subclinical group (3.82%) and the clinical mastitis group (0.98%). *Brochothrix* was found in abundance in the groups with subclinical mastitis (6.03%), clinical mastitis (1.12%), and healthy yaks (6.43%). The percentage abundance of *Lactococcus* in the healthy group was significantly higher (13.03%) than in the other groups. *Carnobacterium* was most abundant in the healthy group (3.56%) but less abundant in the groups with subclinical and clinical mastitis (0.04% and 0.03%, respectively). *Rahnella1* was significantly higher (2.58%) in the healthy group and lower (0.002%) in the clinical mastitis group, but it was not detected in the subclinical mastitis group. Comparing the subclinical mastitis group to the clinical mastitis group, the percentage abundance of *Chryseobacterium* was higher in the mastitis group (1.54%). *Weissella* was also found in the groups with subclinical mastitis (0.23%), clinical mastitis (0.22%), and healthy yaks (0.31%).

The 72 different species of bacteria were identified in each of the three groups at the species level. As shown in Figure 4C, *Pseudomonas fluorescens* (56.93%), *Leuconostoc mesenteroides* (16.14%), *Lactococcus piscium* (12.33%), *Brochothrix thermosphacta* (6.43%), *Carnobacterium maltaromaticum* (3.56%), *Rahnella aquatilis* (2.58%), and *Lactococcus raffinolactis* (0.69%) comprised the majority in the healthy group. There were no indications of *Staphylococcus aureus* or *Streptococcus dysgalactiae* in the healthy group. In the subclinical mastitis group, *Pseudomonas fluorescens* (49.03%), *Moraxella osloensis* (27.94%), and *Psychrobacter cibarius* (12.12%) were the most common pathogens. *Lactococcus piscium* and *Carnobacterium maltaromaticum* abundance were less than 1%. *Lactococcus raffinolactis* and *Rahnella aquatilis* were not identified in the subclinical mastitis group. The species that dominated the clinical mastitis group were *Pseudomonas fluorescens* (77.18%), *Moraxella osloensis* (5.5%), *Psychrobacter cibarius* (11.94%), and *Brochothrix thermosphacta* (1.12%). Comparing the healthy group to the groups with clinical and subclinical mastitis, it was discovered that *Leuconostoc mesenteroides*, *Carnobacterium maltaromaticum*, and *Lactococcus piscium* were present in much higher amounts in the healthy group. *Moraxella osloensis* was found in significantly higher concentrations in the subclinical mastitis group than in the clinical and healthy groups.

**Figure 5:**
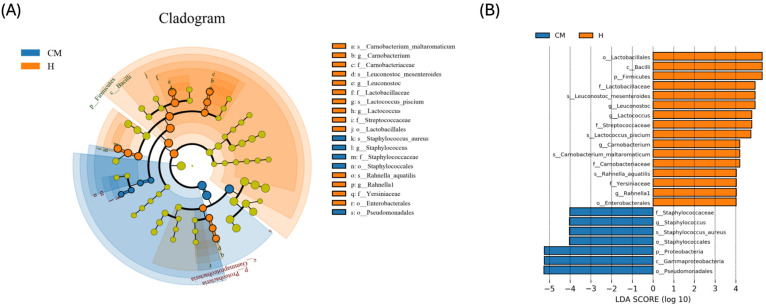
The differentially abundant taxa of healthy (H) yaks and those with clinical mastitis (CM) were identified using linear discriminant analysis effect size (LEfSe) coupled with linear discriminant analysis (LDA) scores. The evolutionary distribution of differentiated biomarkers: (**A**) the cladogram; (**B**) LDA values greater than 4 were the threshold for a meaningful difference.

Since discriminant analysis is not able to detect every taxon, we used LEfSe and LDA to identify the main taxa of differences between groups (Figure 5). Specifically, the LEfSe analysis showed that five genera, four of which were endemic to the healthy group and one of which was unique to the clinical mastitis group—were effective biomarkers to differentiate between the treatment groups. *g-Leuconostoc*, *g-Lactoccus*, *g-Carnobacterium*, and *g-Rahnella 1* were also demonstrated to be diagnostic of the healthy group in addition to these differential taxa. *g-* and *s-Staphylococcus aureus* identified the group with clinical mastitis. In contrast, the group with subclinical mastitis did not exhibit any statistically significant biomarkers.

## 4. Discussion

Mastitis is a significant concern for dairy farmers worldwide because it can result in lower milk yields, higher veterinary expenses, and even financial losses [21]. However, traditional culture-based methods of microbe identification have limitations because they can culture only approximately 1% of the total microbial population and identify microorganisms only at the genus level [22]. This problem can be resolved by using sequencing technologies like PacBio SMRT sequencing to identify and separate the microbiota of healthy yak samples from those suffering from mastitis. The present study identified and analyzed the differences in the bacterial community structure between healthy and mastitis-affected yaks using the full-length 16S rRNA sequence. These advanced techniques enabled a more thorough comprehension of the yak milk microbiota. They can aid in developing efficient mastitis control plans based on the etiological agents of the disease. 

In this study, *Proteobacteria* and *Firmicutes* were the predominant bacterial phyla in the yak milk microbiome across all groups, which varied in relative abundance between milk samples from healthy and mastitis-affected yaks. These results are consistent with previous research on the microbiomes of mastitis in humans and cattle [23,24]. Microbiome diversity measurements indicated differences in microbial dysbiosis between healthy and diseased samples. The current study found significant variations in species richness and microbial diversity between milk samples from healthy yaks and those suffering from mastitis. Furthermore, beta diversity analysis showed that healthy yak milk samples’ microbial composition differed significantly from those affected by mastitis. Although significant differences in alpha diversity were observed between clinical and mastitis samples, some indicators did not show significant differences. These results highlight the complexity and variability of the data.

Clinical mastitis is a common inflammatory disease affecting dairy cattle’s mammary glands. In this study, *Pseudomonas fluorescens* and *Chryseobacterium* were the dominant bacterial species in yaks with clinical mastitis. Gram-negative *Peudomonas fluorescens* is a member of the *Pseudomonadaceae* family of bacteria. It is widely found in nature and is well-known for its capacity to produce a greenish fluorescent pigment [25]. *Pseudomonas fluorescens’* ability to adapt to various ecological settings is one of its noteworthy traits [26]. It is crucial to the bioremediation processes to break down poisons and pollutants found in contaminated environments [27,28]. Its role in synthesizing secondary metabolites is also significant from a pharmacological perspective. It has been found that some strains can manufacture drugs that have antimicrobial or anticancer properties [29]. Although *Pseudomonas fluorescens* exhibits numerous advantageous traits in enhancing plant growth and combating pathogens and abiotic stresses, many species of *Pseudomonas fluorescens* have been associated with human and animal disease [30,31].

Furthermore, *Chryseobacterium* is a less common bacterium that has been linked to several animal diseases [32]. Its presence in clinical mastitis samples raises concerns regarding its potential function as a secondary invader or causative agent in mammary gland infection. Therefore, understanding the microbiological composition associated with clinical mastitis offers important information for creating disease treatment approaches.

Subclinical mastitis is characterized by inflammation of the mammary glands without any outward indications of infection. The quantity and quality of milk produced could decline due to this condition. In our study, the dominant bacterial species found in subclinical mastitis samples were *Moraxella osloensis* and *Psychrobacter cibarius*. *Moraxella osloensis* is a Gram-negative bacterium commonly found in animals and natural environments [32]. *Moraxella osloensis* can colonize human mucosal surfaces as well as skin. Although *Moraxella osloensis* is not commonly associated with clinical infections in humans or animals, it can potentially cause severe infections in immunocompromised individuals or have underlying medical conditions [33,34]. Its presence in subclinical mastitis samples suggests it may have an opportunistic pathogenic impact. Further research may be necessary to understand the specific infection processes and potential health risks. Moreover, *Psychrobacter cibarius* is a psychrophilic bacterial community member characterized by its ability to thrive in cold environments. Numerous cold settings, including soils and refrigerated foods, have isolated this bacterium [35]. Its existence in subclinical mastitis samples suggests adaptation to the udder environment despite its primary association with cold environments. Identifying these prominent bacterial species highlights the complexity of the microbial communities involved in subclinical mastitis. Thus, understanding their prevalence and activity in infected udders may help develop more accurate diagnostic techniques and focused treatment plans for this globally prevalent and economically significant illness affecting dairy cattle. Our results regarding *Psychrobacter* species in yak milk are consistent with those reported by Catozzi et al., who studied the microbiota of buffalo milk during asymptomatic and clinical mastitis and found significant differences in microbiota diversity between healthy and mastitis milk samples. According to their findings, relative abundance was less significant. This comparison highlights the potential impact of mastitis on the microbial composition of milk and supports the relevance of our findings for yak milk [36]. Additionally, Guo et al. studied the antibacterial activity of linalool against *Pseudomonas fluorescens* and highlighted the pathogenic potential of *P. fluorescens* in dairy products, providing a broader context for understanding the presence of *P. fluorescens* in yak milk [37].

*Staphylococcus aureus* is widely acknowledged as a significant causative agent of mastitis, which can significantly alter the composition and quality of milk, posing potential health hazards to consumers [38]. Our study revealed the relative abundance of *Staphylococcus aureus* species in subclinical and clinical mastitis. Persistent or recurrent mastitis may result from *Staphylococcus aureus* bacteria colonizing mammary gland tissue while the infection remains active [39]. These opportunistic bacteria exploit any weakness in the cow’s natural defenses, including teat injury and inadequate cleanliness during milking. Furthermore, studies show that some strains of *Staphylococcus aureus* possess virulence characteristics that enable them to evade the host’s immune system and cause chronic infections [40,41]. This ability prolongs inflammation and prevents the healing process in affected cows. Concerns regarding food safety are raised by identifying *Staphylococcus aureus* in mastitis milk, which further highlights the significance of following reasonable hygiene procedures during the milking and storage process [42]. Contamination can occur through various routes, such as unclean udders or equipment used for milking. This finding suggests that these bacteria may be present in the raw yak milk and their potential to function as opportunistic agents, disrupting metabolic processes, compromising host defense mechanisms, and hindering immunological development, all of which could lead to udder infections of various intensities.

Lactic acid bacteria (LAB) are a diverse group of microorganisms essential to food fermentation and preservation. The dairy industry extensively uses the genus *Lactococcus* because it can be used as a probiotic and in protective and starter cultures [43]. Four distract strains of bacteria were found in our study: *Leuconostoc mesenteroides*, *Carnobacterium maltaromaticum*, *Lactococcus piscium*, and *Lactococcus raffinolactis*. It is interesting to note that fermented foods frequently contain lactic acid bacteria of the type *Leuconostoc mesenteroides*. Numerous *Enterococcus* and *Listeria* species and other opportunistic pathogens are inhibited in their growth by this bacteriocin [44,45]. Extensive research has been conducted on this bacterium because of its potential as a probiotic and for improving food preservation [46,47]. Yak milk demonstrates its potential benefit during the production or storage of milk.

Furthermore, *Carnobacterium maltaromaticum*, a *psychrotrophs* lactic acid bacteria with strain-dependent spoilage and bioprotective characteristics, was also found in the present study [48]. Although their role in food spoiling may be disputed, these bacteria have been connected to beneficial traits such as flavor development during cheese ripening [49]. It has been reported that *Carnobacterium* colonizes the gut in an estrogen-dependent manner and collaborates with other microbes to increase the production of vitamin D in the intestinal tract, activating the host VDR and suppressing colorectal cancer [50].

Most labs are generally recognized as safe microorganisms and crucial for guaranteeing food safety. The *psychrotrophic* foodborne lactic acid bacterium *Lactococcus piscium* is essential to the biopreservation of various food products. This specific species has been found in many food sources, including meat, seafood, vegetables, and dairy products, all typically stored at low temperatures in packaging conditions [51]. The importance of *L. piscium* in food has been highlighted by applying non-cultivatable techniques to the microbial ecosystem characterization process. Food spoilage may be impacted differently by strain variations and different food matrices. Certain strains have been demonstrated to prevent food spoilage and improve sensory quality [52,53].

Furthermore, these beneficial bacteria support antimicrobial activities and raise the food’s safety for consumption by producing organic acids, antimicrobial peptides, and nutrient competition [54]. *L. piscium* shows a wide range of antimicrobial activity against strains of *Salmonella*, *Staphylococcus aureus*, *Clostridium sporogenes*, *Listeria monocytogenes*, and *Escherichia coli* [55,56]. It has been connected to anti-listeria activity dependent on cell-to-cell contact [57].

In addition to their direct impact on food safety, LABs also offer potential health benefits by enhancing digestion and promoting gut health through their ability to hydrolyze lactose and produce enzymes that facilitate nutrient absorption. This study also identified *Lactococcus raffinolactis*, which is widely distributed in various food products and materials derived from plants and animals. It is commonly found in fermented foods, including meat, fish, vegetables, milk, and dairy products [58]. This bacterium is vital to fermentation because of its ability to enzymatically transform complex compounds into simpler forms that promote flavor development [59]. Moreover, it has been found that *L. raffinolactis* is an essential component of several dairy products, such as cheese and yogurt. Its presence significantly affects the taste profiles of these products due to the metabolic activities it goes through during fermentation [60]. Our study highlights the diversity and richness of the microbial communities found in samples of healthy yak milk. Further investigation into these bacterial strains could offer valuable insights into their roles within dairy ecosystems, potentially enhancing food safety and quality control within the dairy industry.

## 5. Conclusions

For this study, we obtained 16S rRNA sequences to understand better the composition and diversity of bacterial communities associated with yak milk in healthy and mastitis-affected individuals. It has been found that the diversity of the milk microbiota has different levels and that the composition of the microbiota differs significantly between healthy yak and mastitis cases. Differential taxonomic abundance patterns within groups can function as indicators of mastitis susceptibility. More research should be conducted to investigate the potential of microbes such as *Leuconostoc mesenteroides*, *Lactococcus piscium*, *Carnobacterium maltaromaticum*, and *Lactococcus raffinolactis* species, which are prevalent in healthy yaks as probiotics for preventing mastitis.

## Data Availability

The original sequence data were submitted to the Sequence Read Archive (SRA) (NCBI, USA) with accession no. PRJNA1130414.

## Supplementary Materials

The following supporting information can be downloaded at: https://www.mdpi.com/article/10.3390/vetsci11080335/s1. Table S1. Summary of the full-length 16S rRNA sequencing data of milk samples in yak. Table S2. The percentage of taxa with top10 relative abundance and corresponding read at various taxonomic levels.
